# Acceptability and feasibility of fitness watches to monitor sleep and physical activity among women living with HIV: broadening the approach to optimizing cardiovascular health

**DOI:** 10.3389/fdgth.2026.1881771

**Published:** 2026-09-03

**Authors:** Kathleen M. Powis, Jessica Lee, Matthew Cole, Debra Riservato, Alexandria DiPerna, Mahboobullah Mirza Baig, Murli Purswani, Natalie Lewis-Vass, Claire A. Berman, Mariam Davtyan, Suzanne Siminski, Paige L. Williams, on behalf of the HOPE Study

**Affiliations:** 1 Departments of Internal Medicine and Pediatrics, Massachusetts General Hospital, Boston, MA, United States; 2 Department of Immunology and Infectious Diseases, Harvard T.H. Chan School of Public Health, Boston, MA, United States; 3 Center for Biostatistics in AIDS Research, Harvard T.H. Chan School of Public Health, Boston, MA, United States; 4 Frontier Science & Technology Research Foundation, Inc., Amherst, NY, United States; 5 BronxCare Health System, Bronx, NY, United States; 6 Department of Epidemiology, Harvard T.H. Chan School of Public Health, Boston, MA, United States; 7 Department of Pediatrics, University of Southern California Keck School of Medicine, Los Angeles, CA, United States; 8 Departments of Biostatistics and Epidemiology, Harvard T.H. Chan School of Public Health, Boston, MA, United States

**Keywords:** acceptabilitity, cardiovascular disease, feasiability, wearable devices, women living with HIV

## Abstract

**Background:**

Women with HIV have increased risk of cardiovascular disease compared to women without HIV. Adopting healthy lifestyle habits in early adulthood, including adequate sleep and physical activity, can mitigate this risk. Fitness watches provide objective measures of sleep and physical activity, and may enable proactive interventions to optimize long-term cardiovascular health. However, the acceptability and feasibility of wearable devices for continuous monitoring of these two measures among women living with HIV has not been adequately explored.

**Methods:**

A pilot acceptability and feasibility study was conducted with thirty women with HIV participating in a US-based prospective observational study. Participants were assigned to one of three fitness watches, two requiring data transfer every 3–5 days, and one without a transfer requirement. Acceptability of using the fitness watch and feasibility of collecting sleep and physical activity data were assessed.

**Results:**

Among the 30 women, median participant age was 36.0 years (Interquartile Range: 30.1, 40.3) with 8 (27%) having > a high school education, and 19 (63%) never having owned a fitness watch. All participants agreed or strongly agreed that the device was easy to use, regardless of device type. Over 80% found it acceptable to collect and share sleep and physical activity data. Only three (10%) participants reported data transfer to be somewhat difficult. Sleep and physical activity data collection was feasible for 90% of devices requiring interim data transfer and 100% of devices without a transfer requirement.

**Conclusions:**

All three fitness watches demonstrated high acceptability and feasibility for continuous data collection among women living with HIV, including participants without prior experience using wearable devices, with minimal concerns regarding privacy or confidentiality. Participants expressed a preference for devices that provided real-time access to personal data, suggesting that user visibility of health metrics may enhance engagement with sleep and physical activity interventions. These findings may inform the design and implementation of wearable device–based interventions to support sleep health and physical activity among women living with HIV of reproductive age.

## Introduction

Women living with HIV (WLHIV) are at increased risk of cardiovascular disease (CVD) compared to women without HIV and men living with HIV (1–8). This elevated risk is multifactorial, reflecting a combination of universal CVD risk factors, HIV-specific mechanisms, and factors unique to WLHIV. Universal risk factors include diabetes mellitus, hypertension, dyslipidemia, obesity, smoking, and family history of early onset CVD (9, 10). Among persons with HIV, chronic inflammation contributes to increased CVD risk, even with sustained HIV-1 viral suppression, a physiological process not captured by traditional CVD risk scores (11–17). Antiretroviral therapy may also increase CVD risk through both direct and indirect pathways, including elevated myocardial infarction risk associated with abacavir, a nucleoside reverse transcriptase inhibitor (18, 19), and metabolic effects such as dyslipidemia with protease inhibitors and weight gain with second generation integrase strand transfer inhibitors (20–24). WLHIV experience heightened CVD risk due to earlier onset of menopause, related to ovarian hormone decline and depletion (2, 25–28).

Currently, lipid-lowering therapy is the only HIV-specific recommendation for primary CVD prevention and is limited to individuals aged ≥40 years with low-to-intermediate 10-year atherosclerotic cardiovascular disease risk (19). These guidelines are informed by the Randomized Trial to Prevent Vascular Events in HIV (REPRIEVE), which demonstrated a 35% reduction in major adverse cardiovascular events over five years among 7,000 adults with HIV at low- to moderate-risk of atherosclerotic cardiovascular disease randomized to pitavastatin, a lipid lowering agent, or placebo (29, 30). While lipid-lowering therapy benefits individuals aging with HIV, opportunities remain to support younger WLHIV across the life course to mitigate long-term CVD risk.

In 2010, the American Heart Association shifted focus from disease management to life-course health promotion, emphasizing the adoption of healthy behaviors early in life to reduce CVD risk (31, 32). The American Heart Association's “Life's Essential 8” framework promotes regular physical activity, healthy sleep, improved diet, smoking cessation, weight management, and control of cholesterol, blood sugar, and blood pressure (32). Optimizing sleep quality and physical activity across the life course of WLHIV represents a tangible strategy to reduce CVD risk. However, intervention development requires acceptable and feasible methods to continuously monitor these behaviors. Fitness watches offer a promising approach, as they can increase physical activity and provide insight into sleep patterns (33–35).

The United States (US)-based Health Outcomes around Pregnancy and Exposure to HIV/ARVs (HOPE) study enrolled over 800 women of reproductive age living with HIV across 14 sites, including 9 states and Puerto Rico. The women enrolled represented a population of specific interest for identification of modifiable lifestyle behaviors, with 51% of the participants reporting poor sleep quality using a validated instrument (36) and over 40% of participants having risk factors for cardiovascular disease including a body-mass-index ≥30 kg/m^2^, a diagnosis of hypertension, type 2 diabetes mellitus, or being a current smoker. To inform an interventional study, we conducted an acceptability and feasibility pilot study to identify the optimal educational materials, level of personal support for use of a fitness watch, and the optimal device for pragmatic use by women living with HIV to achieve robust data collection.

## Materials and methods

### Study population

The HOPE study was a prospective observational study enrolling WLHIV of reproductive age across 14 sites in 9 US states and Puerto Rico (37). Eligible participants were females aged 18 to 45 years with documented HIV infection who were not incarcerated. The HOPE Wearables Pilot study recruited participants from a single HOPE site at BronxCare Health System in New York. Participants were eligible if they expressed interest in or willingness to participate in a wearable device study assessed at entry into the HOPE parent study. The Harvard Longwood Campus Institutional Review Board, the HOPE study's single Institutional Review Board of record, approved the pilot study, and all participants provided written informed consent.

### Fitness watches

The HOPE Wearables Pilot study was designed to assess the acceptability and feasibility of using fitness watches to collect sleep and physical activity data from WLHIV and to compare self-reported measures with device-generated data. Fitness watches were evaluated based on sleep and activity tracking capabilities, durability, cost, battery life, data storage capacity, smartphone compatibility, vendor privacy policies, and availability of application programming interfaces. Devices were categorized as medical research-grade, brand-name consumer devices, or budget fitness watches. Research-grade devices were excluded due to cost, participant burden, or lack of sleep tracking, while budget devices were excluded due to privacy, security and data accessibility concerns. Three brand name fitness watches were selected: the Garmin Vivosmart 5, Garmin Vivofit 4, and the Fitbit Inspire 3. All devices had available application programming interfaces and did not require Apple Health or Google Fit for data retrieval. Vendors also demonstrated ongoing maintenance of firmware and mobile applications.

The Vivosmart 5 and Inspire 3 featured sleep and activity tracking, heart rate monitoring, and accelerometers, and required synchronizing every 5–7 days for data transfer. The Vivofit 4 watch tracked sleep and physical activity via accelerometry but lacked heart rate monitoring, allowing extended battery life and data storage without requirement for interim data synchronization. Data from the Vivofit 4 were retrieved at the final study visit. Sleep and activity data were viewable on the Vivosmart 5 and Inspire 3 watches, but this was not an option with the Vivofit 4.

The HOPE Wearables Pilot Study aimed to enroll 30 participants, with 10 participants randomly assigned to each device using permuted blocks in a 1:1:1 fashion. Participants received a wearables study package containing the assigned fitness watch, wristbands of different sizes, a tablet for self-reporting sleep and physical activity over 10 days during the 30-day study period, charging equipment, and device-specific training materials. The kits also included a paper log (Supplementary 1) for self-reporting sleep and physical activity for participants who did not wish to use the tablet, a training guide (Supplementary 2), and a frequently asked questions document (Supplementary 3), the latter two of which were customized to the specific fitness watch. Contents of the package were developed in coordination with HOPE Community Advisory Board members, who also provided input on clarity and style of the information guides.

### Study activities

At enrollment, participants provided updated sociodemographic information, reported prior experience with fitness watches, and described their internet accessibility. Participants received training on device use and, when applicable, data transfer procedures. Specifically, with the Inspire 3 and the Vivosmart 5, data transfer required periodic synchronizing with the supplied tablet. Study staff contacted participants at four intervals (days 2–4, 5–7, 12–17, and 24–28) during the 30-day study period to provide support, monitor device issues, and facilitate completion of self-reported measures between days 10 and 19, and to schedule a concluding in-person visit interview and retrieve wearables study package materials. During the final study visit, participants completed an online survey assessing acceptability of device use, feasibility of data transfer (when applicable), perceived impact on sleep and physical activity, concerns regarding privacy or HIV status disclosure, and clarity of study materials. While the informed consent form approved by the Institutional Review Board included a statement about one aspect of the pilot, specifically that it was being conducted to understand how practical it would be to have a study participant wear a fitness watch night and day for a 30-day period, the online survey also inquired about whether the participant had worn the device for more, the same, or less time than expected when they initially opted to enroll in the study.

### Statistical analyses

Descriptive statistics were used to summarize participant characteristics by fitness watch, using medians and interquartile ranges (IQR) for continuous variables and counts with proportions for categorical or ordinal variables. User responses to 5-point Likert Scale questions on acceptability and ease of using a fitness watch were quantified with options ranging from strongly agree to strongly disagree. “Yes/No” questions about feelings surrounding real-time access to sleep and physical activity data for the two devices with viewable data was quantified as counts and proportions. Measurements of sleep from the device, including hours of sleep [overall, light sleep, rapid eye movement (REM), and deep sleep], minutes awake, and sleep efficiency (Inspire 3 only) were summarized by fitness device using means and standard deviations (SDs). Activity measures from the device including minutes of activity (total, vigorous and moderate), resting heart rate (Inspire 3 and Garmin 5), daily steps, and daily distance were also summarized by device using means and SDs.

Self-reported sleep measures collected over a 10-day period were compared with fitness watch-derived data. For each of the 10 days, the difference in number of hours spent sleeping and number of minutes awake during their sleep period between self-report and the watch was obtained. The average difference over the 10 days for each participant was calculated, with positive values reflecting a higher amount of self-reported time spent sleeping (or minutes awake after initially falling asleep and before completing the sleep periods) compared to the watch. Means of these average within-participant differences were summarized by fitness watch. To visually evaluate the agreement in the self-reported hours of sleep compared to the watch data, a Bland-Altman plot was created with a separate point indicating the difference for each day of participant self-report compared to concomitant fitness watch use (38). Self-reported moderate and strenuous physical activity measures were recorded by the participant on each of the 10 days for which they completed a log using categorical responses, rather than exact times. For example, daily moderate activity was reported as none, <30 min, 30 min to <1 h, 1 to <2 h, 2 to <4 h, or 4 h or more. However, the participants were not asked to report on minutes of light activity. The absence of reporting on light activity and the use of categorical responses for moderate and strenuous activity precluded comparison of activity between self-report and the devices. All analyses were performed using SAS software, Version 9.4 (SAS Institute Inc., Cary, NC, USA).

## Results

Between November 2024 and March 2025, 30 HOPE participants were approached about the HOPE Wearables Pilot Study, and all consented to study participation. Median age of participants was 36.0 years (IQR 30.1, 40.3), with the 10 participants assigned to the Vivosmart 5 device being younger, on average, than the other two groups, at a median age of 33.0 (IQR 26.9, 36.2) (Table 1). Twenty participants (67%) self-identified as being Black or African American and 23% as white, with small numbers in other racial groups, similar to the distribution in the parent HOPE study. Seven participants (23%) had acquired HIV perinatally. Six (20%) participants had less than a high school education, 7 (23%) reported currently being in school, and 17 (57%) were not employed at time of enrollment. Five (16%) participants reported currently parenting at least one child ≤5 years of age and 19 (63%) participants indicated that they had never owned a fitness watch.

**Table 1:** Participant characteristics at study entry by type of fitness watch.

Characteristics	Assigned fitness watch			Total (*N* = 30)
	Inspire 3 (*N* = 10)	Vivofit 4 (*N* = 10)	Vivosmart 5 (*N* = 10)	
Median age (IQR)	36.9 (31.1, 43.3)	38.7 (30.6, 43.4)	33.0 (26.9, 36.2)	36.0 (30.1, 40.3)
Race				
Black/African American	6 (60%)	8 (80%)	6 (60%)	20 (67%)
White	2 (20%)	2 (20%)	3 (30%)	7 (23%)
Native American	0 (NA)	0 (NA)	1 (10%)	1 (3%)
More than one race	2 (20%)	0 (NA)	0 (NA)	2 (7%)
Mode of HIV acquisition				
Perinatal	2 (20%)	2 (20%)	3 (30%)	7 (23%)
Non-perinatal	8 (80%)	8 (80%)	7 (70%)	23 (77%)
Highest level of educational attainment				
Less than high school	2 (20%)	2 (20%)	2 (20%)	6 (20%)
High school diploma or GED	6 (60%)	4 (40%)	6 (60%)	16 (53%)
More than high school	2 (20%)	4 (40%)	2 (20%)	8 (27%)
Currently attending school	4 (40%)	3 (30%)	0 (NA)	7 (23%)
Currently working	4 (40%)	5 (50%)	4 (40%)	13 (43%)
Currently parenting at least one child ≤5 years-of-age	1 (10%)	1 (10%)	3 (30%)	5 (17%)
Ever owned a fitness watch	5 (50%)	2 (20%)	4 (40%)	11 (37%)

GED, general educational development; HIV, human immunodeficiency virus; IQR, interquartile range.

All 30 participants, regardless of fitness watch assignment, either agreed or strongly agreed that the fitness watch was easy to use, and all indicated willingness to participate in a future study using a fitness watch (Table 2). Overall, 13 (43%) participants indicated wearing the fitness watch for more time than they initially expected, while 15 (50%) reported wearing the device for about the same amount of time as they expected. In total, 26 (87%) participants agreed or strongly agreed that the watch helped them keep better track of their sleep, while all 30 agreed or strongly agreed that the watch enabled them to keep better track of their physical activity, despite the fact that those using the Vivofit 4 could not view their data in real-time (Table 2). Only two (7%) participants reported that wearing the fitness watch raised concern about risk of disclosure of their HIV status to others. All participants agreed or strongly agreed that the instructions were clear, regardless of assigned device. Among the 20 participants assigned to devices requiring periodic data transfer, 17 (85%) reported the process as very easy, 1 (5%) as somewhat easy, and 2 (10%) as somewhat difficult. None of the participants reported internet-related challenges, and only one participant reported technical issues with the tablet during data transfer.

**Table 2:** Acceptability and feasibility by fitness watch and tablet use by device and overall.

	Assigned fitness watch			Total (*N* = 30)
	Inspire 3 (*N* = 10)	Vivofit 4 (*N* = 10)	Vivosmart 5 (*N* = 10)	
The fitness watch was easy to use				
Strongly agree	6 (60%)	5 (50%)	6 (60%)	17 (57%)
Agree	4 (40%)	5 (50%)	4 (40%)	13 (43%)
Would sign up for a study using a fitness watch again				
Yes	10 (100%)	10 (100%)	10 (100%)	30 (100%)
Wore fitness watch for more or less time than expected, on average				
More than I expected	4 (40%)	4 (40%)	5 (50%)	13 (43%)
About the same as I expected	5 (50%)	5 (50%)	5 (50%)	15 (50%)
Less than I expected	1 (10%)	1 (10%)	0 (0%)	2 (7%)
Liked having the fitness watch to help me keep better track of my sleep				
Strongly agree	5 (50%)	1 (10%)	5 (50%)	11 (37%)
Agree	3 (30%)	7 (70%)	5 (50%)	15 (50%)
Neither agree or disagree	2 (20%)	1 (10%)	0 (0%)	3 (10%)
Disagree	0 (0%)	1 (10%)	0 (0%)	1 (3%)
Liked having the fitness watch to help me keep better track of my physical activity				
Strongly agree	5 (50%)	1 (10%)	5 (50%)	11 (37%)
Agree	5 (50%)	9 (90%)	5 (50%)	19 (63%)
Feel that I changed my sleep because of wearing the fitness watch				
Strongly agree	4 (40%)	0 (0%)	0 (0%)	4 (13%)
Agree	0 (0%)	0 (0%)	2 (20%)	2 (7%)
Neither agree or disagree	4 (40%)	3 (30%)	5 (50%)	12 (40%)
Disagree	2 (20%)	5 (50%)	3 (30%)	10 (33%)
Strongly disagree	0 (0%)	2 (20%)	0 (0%)	2 (7%)
Feel that I changed my physical activity because of wearing the fitness watch				
Strongly agree	2 (20%)	0 (0%)	0 (0%)	2 (7%)
Agree	1 (10%)	3 (30%)	3 (30%)	7 (23%)
Neither agree or disagree	4 (40%)	2 (20%)	5 (50%)	11 (37%)
Disagree	2 (20%)	5 (50%)	2 (20%)	9 (30%)
Strongly disagree	1 (10%)	0 (0%)	0 (0%)	1 (3%)
Wearing the fitness watch for a study made me feel concerned about risk of disclosure of my HIV status				
Agree	1 (10%)	0 (0%)	1 (10%)	2 (7%)
Neither agree or disagree	2 (20%)	0 (0%)	1 (10%)	3 (10%)
Disagree	6 (60%)	8 (80%)	5 (50%)	19 (63%)
Strongly disagree	1 (10%)	2 (20%)	3 (30%)	6 (20%)
The written instructions for using the fitness watch were clear				
Strongly agree	7 (70%)	4 (40%)	7 (70%)	18 (60%)
Agree	3 (30%)	6 (60%)	3 (30%)	12 (40%)
Received clear verbal guidance from the site staff				
Strongly agree	9 (90%)	5 (50%)	8 (80%)	22 (73%)
Agree	1 (10%)	5 (50%)	2 (20%)	8 (27%)
Fitness watch broke or stopped working at any time during study				
No	10 (100%)	10 (100%)	9 (90%)	29 (97%)
Yes	0 (0%)	0 (0%)	1 (10%)	1 (3%)
The tablet was easy to use				
No	2 (20%)	0 (0%)	1 (10%)	3 (10%)
Yes	8 (80%)	1 (10%)	9 (90%)	18 (60%)
I did not use a tablet	0 (0%)	9 (90%)	0 (0%)	9 (30%)
Tablet broke or stopped working at any time during study				
No	10 (100%)	10 (100%)	10 (100%)	30 (100%)
Ease of transferring data from fitness watch^1^				
Very easy	9 (90%)	NA	8 (80%)	17 (85%)
Somewhat easy	0 (0%)	NA	1 (10%)	1 (5%)
Somewhat difficult	1 (10%)	NA	1 (10%)	2 (10%)
Trouble transferring data due to technical issues with the watch^1^				
No	10 (100%)	NA	10 (100%)	20 (100%)
Trouble transferring data due to technical issues with the tablet^1^				
No	10 (100%)	NA	9 (90%)	19 (95%)
Yes	0 (0%)	NA	1 (10%)	1 (5%)
Trouble transferring data due to technical issues with network connection^1^				
No	10 (100%)	NA	10 (100%)	20 (100%)
Trouble transferring data due to taking too much time^1^				
No	10 (100%)	NA	10 (100%)	20 (100%)

1 Use of tablet for data transfer from the fitness watch was not necessary for the Vivofit 4.

Among the 20 participants with real-time access to their data, 14 (70%) reported positive feelings about viewing their data and 7 (35%) reported feeling motivated by data access (Table 3). Only one participant reported anxiety related to viewing these data. Among the 10 participants assigned to a device without display data, 6 (60%) expressed disappointment about not having access to their data.

**Table 3:** Participant feelings about real-time access to sleep and physical activity data.

Question	Assigned fitness watch		Total (*N* = 20)
	Inspire 3 (*N* = 10)	Vivosmart 5 (*N* = 10)	
Felt positive about having access to data on the fitness watch	6 (60%)	8 (80%)	14 (70%)
Felt motivated about access to data on the fitness watch	4 (40%)	3 (30%)	7 (35%)
Felt anxious about having access to data on the fitness watch	1 (10%)	0 (0%)	1 (5%)
Felt depressed about having access to data on the fitness watch	0 (0%)	0 (0%)	0 (0%)
Felt worried about having access to data on the fitness watch	1 (10%)	0 (0%)	1 (5%)

**Figure 1:**
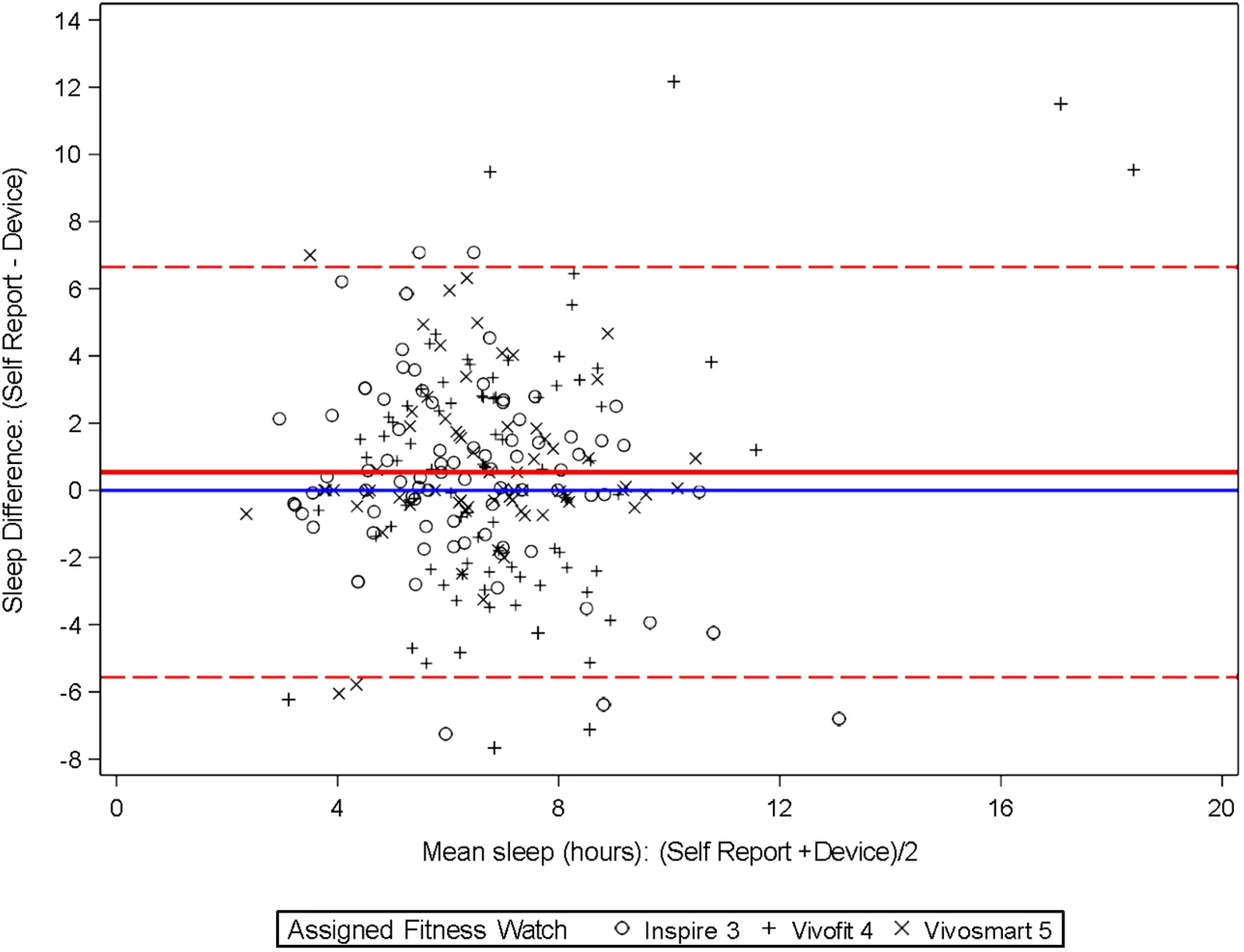
Bland-Altman plot of agreement in hours of sleep between participant self-report and fitness watch data. Each point on the plot above reflects the difference in hours between self-report and the device for up to 10 days per participant versus the mean hours of sleep from these two measures to appropriately scale differences. The red line shows the overall mean difference between self-report and the device hours of sleep from these two measures. If agreement is good, most points should appear close to 0 and 95% of the points should fall within 2 SDs of the mean difference.

A total of 28 (93%) participants provided self-reported data over 10 days while concurrently wearing a fitness device. On average, self-reported sleep duration exceeded device-measured sleep by just over half an hour (mean difference: 0.56 h; Table 4). Participants reported waking a mean of 1.27 times after sleep onset, with fewer minutes awake during these periods compared with device estimates (mean 12.3 vs. 33.8 min, respectively), yielding an average difference of −20.4 min. Participants wearing the Inspire 3 exhibited smaller mean differences between self-reported and device-measured sleep duration compared with the other two devices. Across devices, mean nightly sleep comprised 226 min of light sleep, 84 min of deep sleep, and 39 min of REM sleep, with deep sleep accounting for 22% of total sleep. Agreement between self-reported and device-measured sleep duration is illustrated in the Bland-Altman plot (Figure 1), with the mean difference of approximately 0.5 h (red line) indicating slightly longer sleep duration by self-report than by device measurement.

**Table 4:** Summary of self-reported data and fitness watch data on sleep measures.

Sleep characteristics	Assigned fitness watch			Total (*N* = 30)
	Inspire 3 (*N* = 10)	Vivofit 4 (*N* = 10)	Vivosmart 5 (*N* = 10)	
Mean hours of sleep (SD)				
Self-report	6.50 (1.79)	7.66 (2.42)	7.05 (1.68)	7.07 (1.98)
Fitness watch data^1^	6.14 (2.07)	6.75 (1.08)	6.31 (1.00)	6.39 (1.46)
Mean difference in hours of sleep (SD)^1^^,^^2^	+0.32 (1.52)	+0.68 (2.76)	+0.69 (1.47)	+0.56 (1.92)
Mean minutes awake (SD)^3^				
Self-report	13.5 (10.8)	11.8 (11.2)	11.7 (6.9)	12.3 (9.5)
Fitness watch data^1^	39.1 (22.3)	43.8 (25.1)	17.9 (11.5)	33.8 (22.8)
Mean difference in minutes awake (SD)^1^^,^^2^	−23.3 (29.2)	−31.3 (27.5)	−6.4 (6.5)	−20.4 (25.1)
Mean minutes of light sleep (SD)^4^	169.6 (97.9)	267.2 (48.0)	247.3 (50.6)	226.0 (80.8)
Mean percent of total sleep that is light	48.5 (20.4)	67.2 (5.1)	64.7 (5.3)	59.7 (15.1)
Mean minutes of deep sleep (SD)^4^	47.2 (26.6)	137.9 (28.7)	70.2 (14.3)	83.8 (45.5)
Mean percent of total sleep that is deep	13.4 (6.2)	32.8 (5.1)	20.0 (6.3)	21.8 (10.0)
Mean minutes of REM sleep (SD)^4^	53.5 (32.2)	NA	61.3 (26.4)	57.2 (29.1)
Mean percent of total sleep that is REM	19.6 (4.9)	NA	16.8 (6.5)	18.2 (5.8)
Mean hours in bed (SD)^4^	7.09 (2.39)	NA	NA	7.09 (2.39)
Mean sleep efficiency^5^ (SD)^4^	87.3 (3.0)	NA	NA	87.3 (3.0)

1 Only 28 participants concurrently completed the sleep and physical activity log (self-report) with concurrent use of the fitness watch, including 10 using the Inspire 3, and 9 each using the Vivofit 4 and Vivosmart 5.

2 Positive values for mean differences reflect a higher self-reported value compared to fitness watch data, whereas negative values reflect a lower self-reported value compared to fitness watch data.

3 Mean minutes awake refers to the number of minutes where participants had awoken after initially falling asleep and before they completed the period of sleep.

4 Data from fitness watch only. The Vivofit 4 did not record REM sleep time. Only the Inspire 3 recorded total time in bed and therefore, sleep efficiency could only be calculated for this device.

5 Sleep Efficiency is calculated by using fitness watch data of hours in bed as the denominator and hours of sleep as the numerator × 100.

Abbreviations: REM, rapid eye movement; SD, standard deviation.

Devices recorded a mean of 175 min of total daily activity, 4 min of vigorous activity, and 106 min of moderate activity (Supplementary Table 1). Participants wearing an Inspire 3 were noted to have higher average total activity compared with those using the Vivofit 4 and Vivosmart 5 devices (293 vs. 61 and 171 min, respectively), and on average also took a higher number of steps each day (9,019 vs. 5,777 and 7,477) and traveled a longer distance (3.6 miles vs. 2.3 and 3.1 miles). Mean resting heart rate was 66 beats per minute and was similar between the two devices that captured heart rate data, the Inspire 3 and Vivosmart 5 devices.

## Discussion

Fitness watches are widely adopted for personal use and are increasingly being used in research to monitor and promote sleep quality and physical activity (39–41). In this pilot study, fitness watch use for 30-day monitoring of sleep and physical activity was highly acceptable among WLHIV of reproductive age, and data transfer was feasible for 90% of participants using devices requiring synchronization. Across devices, self-reported sleep duration was modestly longer than device-measured sleep, while self-reported wake time after sleep onset was shorter than device estimates. Only two participants reported concern about potential disclosure of HIV status related to wearing a fitness watch.

The high acceptability observed in this pilot study likely reflects several key design and implementation strategies. First, extensive collaboration with the HOPE Community Advisory Board informed study development, with particular attention to potential concerns such as inadvertent HIV disclosure and barriers related to internet access, allowing these issues to be addressed proactively. For example, wearables kits were not branded with the HOPE study logo, and educational materials focused exclusively on device and tablet use. Second, device-specific training materials and frequently asked questions were developed and independently tested to ensure clarity and usability. Third, routine participant check-ins provided opportunities for additional instruction, troubleshooting, and reminders regarding study procedures. Together, these operational strategies likely contributed to the high participant study acceptability and feasibility. However, two (7%) participants did express concern about potential disclosure of their HIV status by wearing the fitness watch, underscoring the need to expand the study's “Frequently Asked Questions” document with suggested responses for inquiries and to provide participants with discreet, standardized explanations to address inquiries by others about the device.

Sleep duration of 7–8 h per 24 h period promotes health and wellbeing, optimizes attention, and supports learning and memory (42–44). Ideally, approximately 25% of total sleep time is spent in deep sleep, the most restorative stage of the sleep cycle, supporting tissue repair, bone formation, and immune function (45, 46). In this study, mean sleep duration measured by the fitness watches was below the recommended threshold at 6.39 h, with 22% of sleep spent in deep sleep. However, substantial variability was observed across devices. These findings highlight opportunities to improve sleep health using wearable devices, which provide objective estimates of sleep duration and stages, metrics often overestimated or not captured through self-report, while also underscoring the need to account for inter-device variability when interpreting results.

It is unclear why the Inspire 3 device provided higher average activity compared to the other two devices. Given the relatively limited sample sizes, it could be that the women assigned to the Inspire 3 did have higher activity levels on average, or that the Inspire 3 was more sensitive than the other two devices, capturing less vigorous activity compared to the other wearable devices. Differences in proprietary activity-detection algorithms, sensor characteristics, activity-minute definitions, wearer compliance, and other device-specific measurement characteristics have been previously reported (47, 48). Therefore, the observed differences may reflect, at least in part, variation in how activity was measured across devices.

Although this pilot study demonstrated that fitness watch use was acceptable and feasible, even among participants without prior experience, several limitations should be noted. Participants were recruited from among WLHIV who had already expressed interest in a wearable device study, potentially inflating acceptability estimates. Indeed, in the parent HOPE study, 23% of 660 participants reported unwillingness to participate in a wearable device study (49). Acceptability and feasibility may therefore be lower in the broader population of WLHIV. Access to reliable internet for data transfer may also limit participation in larger studies. Additionally, given the small sample size and that all study materials were provided in English, the findings may not be generalizable to non-English-speaking populations. While self-reported sleep could be compared with device-derived measures, the structure of the physical activity questions precluded direct comparison with device data; however, this was not a primary objective of the pilot. Additionally, the Vivofit 4 device lacked real-time heart rate activity, which would preclude use in a interventional study whose design relied, in whole or in part, on immediate participant feedback to improve activity.

WLHIV face a disproportionately higher risk of cardiovascular disease compared to both women without HIV and men living with HIV (1–8). The American Heart Association advocates a life-course approach to cardiovascular health, emphasizing early adoption of behaviors such as adequate sleep, regular physical activity, healthy diet, smoking cessation, weight management, and control of cholesterol, blood sugar, and blood pressure (31, 32). For WLHIV, this elevated risk necessitates a shift from siloed HIV care to integrated models that embed cardiovascular prevention at HIV diagnosis and throughout HIV treatment, particularly for younger women. Wearable devices, such as fitness watches, offer a practical approach to support this shift by providing objective, real-time measures of sleep and physical activity, key modifiable drivers of cardiovascular health, thereby enabling more proactive, data-informed care in partnership with WLHIV. Additionally, integrating wearable device data into routine clinical workflows, such as electronic health records and patient monitoring systems, could enable clinicians to track behavioral risk factors longitudinally, identify early deviations, and deliver timely, personalized interventions.

## Conclusions

WLHIV of reproductive age found participation in this wearable device-based study to be both acceptable and feasible. Participants preferred devices that allowed real-time data access, suggesting that visibility of personal metrics may enhance engagement in interventions targeting sleep and physical activity. These findings may inform the design and implementation of wearable device–based interventions to support sleep health and physical activity among women living with HIV of reproductive age.

## Data Availability

The raw data supporting the conclusions of this article will be made available by the authors, without undue reservation.

## HOPE study

The following institutions, clinical site investigators and staff participated in conducting the HOPE study, in alphabetical order: Ann & Robert H. Lurie Children's Hospital of Chicago: Jennifer Jao, Lela Lartey, Kathleen Malee; Baylor College of Medicine: Mary Paul, Alejandra Martinez, Lynnette Harris; BronxCare Health System: Murli Purswani, Martha Cavallo, Mahboobullah Mirza Baig, Alma Villegas-Schwalenberg; Children's Diagnostic & Treatment Center: Lisa-Gaye Robinson, Kierra Archer, Alan Bernegger, Patricia Garvie; Jacobi Medical Center: Andrew Wiznia, Marlene Burey, St. Jude Children's Research Hospital: Katherine Knapp, Chloe Burkhead, Gheri Terry, Megan Wilkins; State University of New York Downstate: Stephan Kohloff, Ava Dennie; Tulane University School of Medicine: Margarita Silio, Dornese Jones, Medea Gabriel, Patricia Sirois; University of Alabama, Birmingham: Cecelia Hutto, Paige Hickman, Dan Marullo; University of Colorado, Denver: Elizabeth McFarland, Carrie Chambers, Robin McEvoy; University of Florida, Center for HIV/AIDS Research, Education and Service: Mobeen Rathore, Saniyyah Mahmoudi, Staci Routman; University of Miami: Rana Chakraborty, Lorena Bracho, Anai Cuadra; University of Puerto Rico School of Medicine, Medical Science Campus: Zoe M. Rodriguez, Lizmarie Torres, Nydia Scalley; University of Southern California: Toinette Frederick, Mariam Davtyan, Cristina Hernandez, Guadalupe Morales Avendano.
