# Supplementary material for: Acceptability and feasibility of fitness watches to monitor sleep and physical activity among women living with HIV: broadening the approach to optimizing cardiovascular health
Source: Front Digit Health. 2026 Sep 3;8:1881771. doi: 10.3389/fdgth.2026.1881771 (PMC13582592; doi:10.3389/fdgth.2026.1881771)

Supplement 1: First Day of Sleep and Physical Activity Paper Log

Sleep and Physical Activity Log

Welcome to your Sleep and Physical Activity Log for the Wearables Pilot Study. Please fill out this log daily during the 10-day window starting on the day the site study staff told you start. You will be receiving a reminder from the site study staff about completing this log. **At the top of the columns, please enter the day of the week and the date for the sleep and physical activity data you are reporting (the site staff may have filled this in for you).** Please remember that filling out this log is your choice. If you do not wish to fill it out or your schedule does not allow you to complete the log, we understand. However, for every day that you complete the log in the 10-day window that you have been asked to complete the log, you will be compensated $10 per day.

INSTRUCTIONS:
Please record dates, using the format (month)/(day)/(year).
Please enter times, using the format (hour):(minute) and indicate by circling AM or PM.
Please record data about your daily sleep in this first section.

**DAY 1:** Day of Week: ________________ Date: _____/_____/_______	
**Sleep**	
What time did you go to sleep?	_____:_____ AM / PM
How long did it take you to fall asleep:	______ minutes
What time did you wake up?	_____:_____ AM / PM
Total hours of your actual sleep *(This may be different than the number of hours you spend in bed.):*	______ hours ______ minutes
How many times did you wake up in the middle of your sleep?	______ times
Based on how many times you awoke during your sleep; please share the total amount of time you were awake before going back to sleep. For example, if you woke up twice and the first time you were awake for about 15 minutes before falling back to sleep and the second time you were awake for 30 minutes before falling back to sleep, you would report 45 minutes. If you said "0" wake ups in the previous question, then enter "0" here.	______ minutes
Number of naps you took yesterday:	______ times
Total naptime yesterday (If you did not nap, enter "00" for hours and minutes):	______ hours ______ minutes
Is there anything that affected your sleep that you would like to share?	

**DAY 1:** Day of Week: ________________ Date: _____/_____/_______	
**Physical Activity**	
Think of your day. How much of your day did you spend doing moderate or vigorous activity either while at home, at work, or doing activities outside of your home?<br>*Please describe your activity each day by writing in text. Examples include walking, playing a sport, dancing, lifting weights, working in an environment where heavy lifting or other physically demanding activities are required, etc.*	
Total duration of <u>moderate</u> activity each day:<br><br>Moderate activity includes brisk walking,<br>bicycling, vacuuming, gardening, or anything else that causes some increase in breathing or heart rate	___ None<br>___ < 30 minutes<br>___ 30 minutes to < 1 hour<br>___ 1 hour to < 2 hours<br>___ 2 hours to < 4 hours<br>___ 4 hours or more
Total duration of <u>vigorous</u> activity each day:<br><br>Vigorous activity running, aerobics,<br>heavy yard work, or anything else that causes large increases in breathing or heart rate	___ None<br>___ < 30 minutes<br>___ 30 minutes to < 1 hour<br>___ 1 hour to < 2 hours<br>___ 2 hours to < 4 hours<br>___ 4 hours or more
**Fitness Device**	
How long did you wear your watch today:	______ hours ______ minutes
How long was your watch charging today, if at all *(If the watch was not charging, enter 00 for hours and minutes.)*:	______ hours ______ minutes

Supplement 2: Training Guides for Use of Fitness Watch and Tablet

4. You should see the battery icon in the middle of the tablet screen.
5. Charge the tablet to at least 80% before continuing.
6. Once the tablet is at least 80% charged, press and hold the Power button for approximately 3-5 seconds or until you see the Lenovo logo on the screen.
7. When the Lenovo logo appears, let go of the Power button.
8. Allow the tablet to turn on. After a few seconds, the home screen will be displayed. A message at the top will say, Unlock for all features and data.
9. With your index finger, swipe up from the bottom of the tablet to open the PIN pad. Enter the PIN listed in the login credentials leaflet and then tap the right pointing arrow (that is next to the zero).
10. After a few seconds, you will see the Home screen with the Fitbit app.

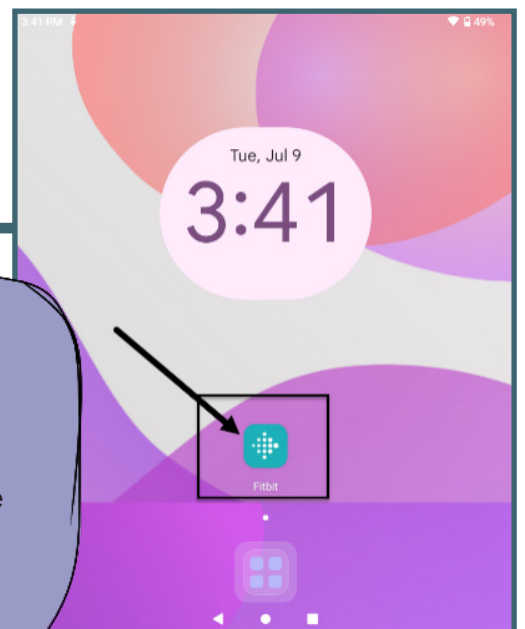

NOTE: For your privacy, the tablet is configured to go into sleep mode after one minute of inactivity. Press once on the power button to "wake up" the tablet and reenter the PIN.

STEP 3. Connect the tablet to Wi-Fi

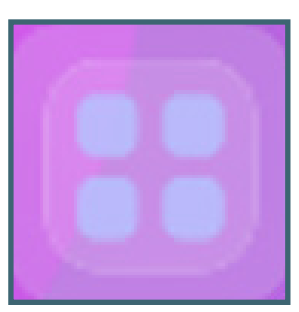

1. Tap the icon which looks like a box with four boxes in it located at the bottom of the Home screen.
2. Tap settings
3. Tap Network & Internet
4. Tap Internet. Locate the Wi-Fi network and connect to it.

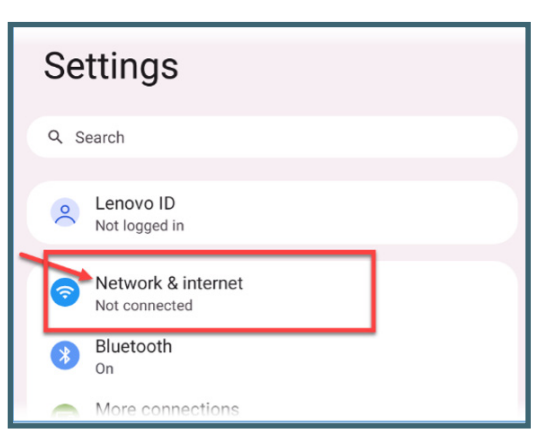

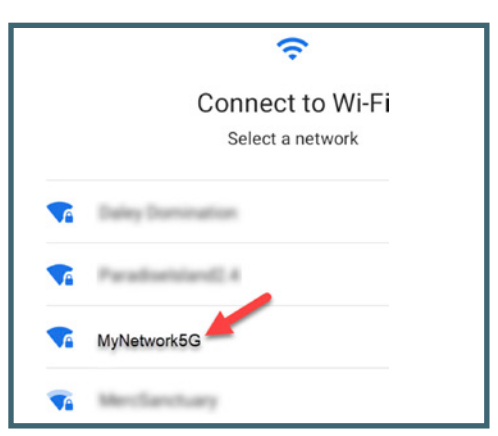

5. Once connected, tap the square icon at the bottom of the screen and tap Clear all.

STEP 6. SELECT YOUR WEARING WRIST

1. Open the Fitbit app
2. Tap on the wearable icon in the upper left corner
3. Under Devices, tap the inspire 3 banner.
4. The inspire 3 screen will open.
5. Scroll down to Wrist Placement
6. Choose the wrist on which you will be wearing the device (Dominant or Non-Dominant)
7. The change will be saved automatically.
8. Tap the back arrow in the upper left corner to go back to the Devices screen
9. Tap the back arrow in the upper left corner to go back to the Today screen.

CONGRADUALTIONS!

You've completed all the setup steps.

YOU MAY NOW BEGIN WEARING THE FITBIT INSPIRE 3.

Please now review the Inspire 3 Usage Guide and FAQ/Troubleshooting guide which are in your kit. These will provide more detail on how to use the Fitbit throughout the study.

FITBIT INSPIRE 3

GETTING STARTED

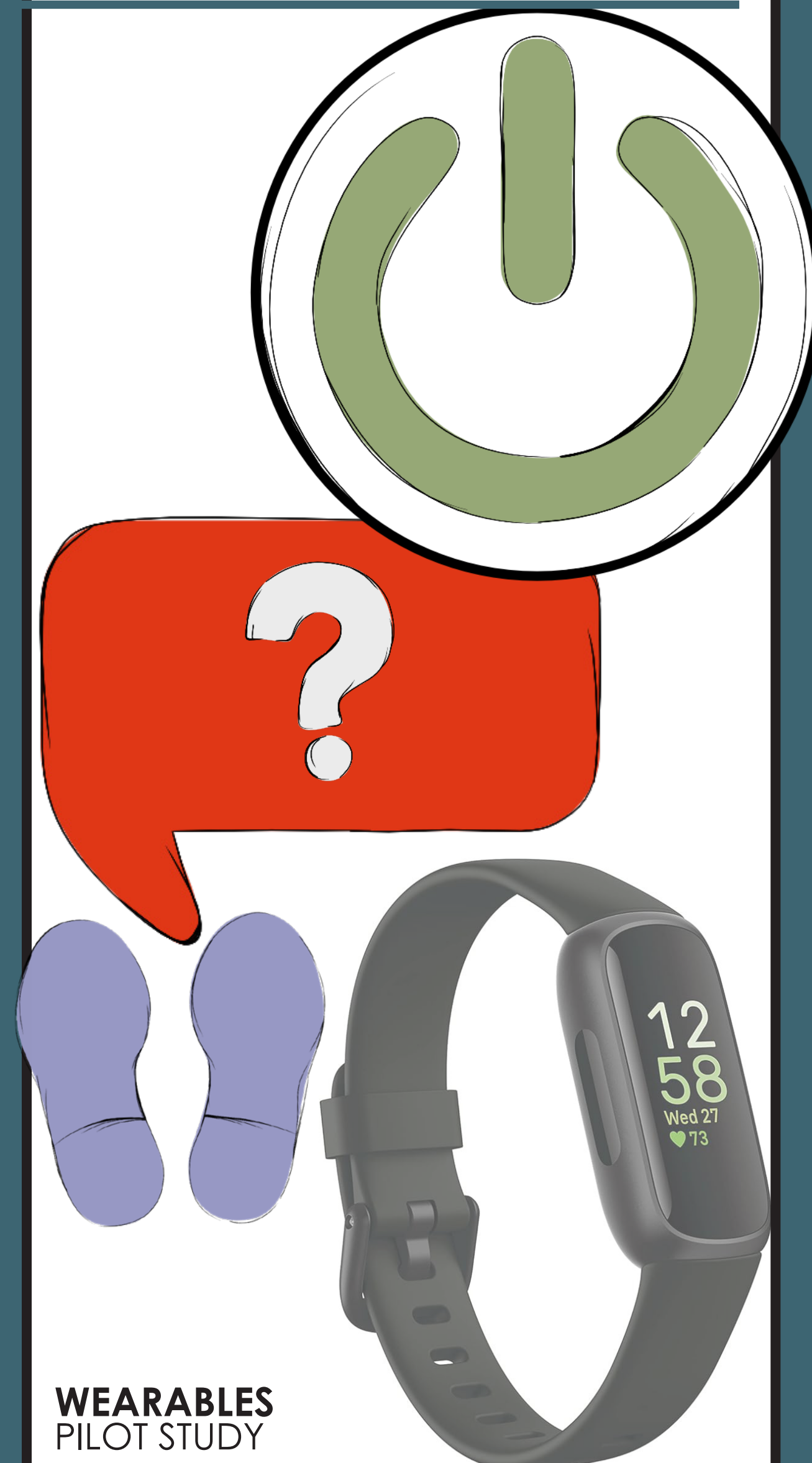

WEARABLES
PILOT STUDY

Your enrollment kit contains the following devices, accessories, and literature:

- One Fitbit Inspire 3, a large and small wrist band, a charging cable
- One Lenovo tablet and charging cable
- One charging brick
- A leaflet with the login credentials for your kit
- the Fitbit Inspire 3 getting started manual (this brochure)
- the Fitbit Inspire 3 usage guide
- The Fitbit Inspire 3 FAQ and troubleshooting guide

Before you can begin wearing the device, you must complete the following steps in the order listed.

STEP 1. Charge the Fitbit Inspire 3

- The Fitbit inspire 3 will be on when you receive it, but it will need to be charged.
- Hold the end of the charging cable near the port on the back of the tracker until it attaches magnetically.
- Make sure the pins on the charging cable align with the port.
- The connection is secure when the tracker vibrates and a battery icon appears on the screen.
- Plug the other end of the cable into a UPS port of the charging brick provided. Plug the brick into the wall.

STEP 2. Start and charge the tablet

1. Unbox the tablet.
2. Plug the tablet in to charge it using the cable provided.
3. After it is plugged in, press once on the Power button.

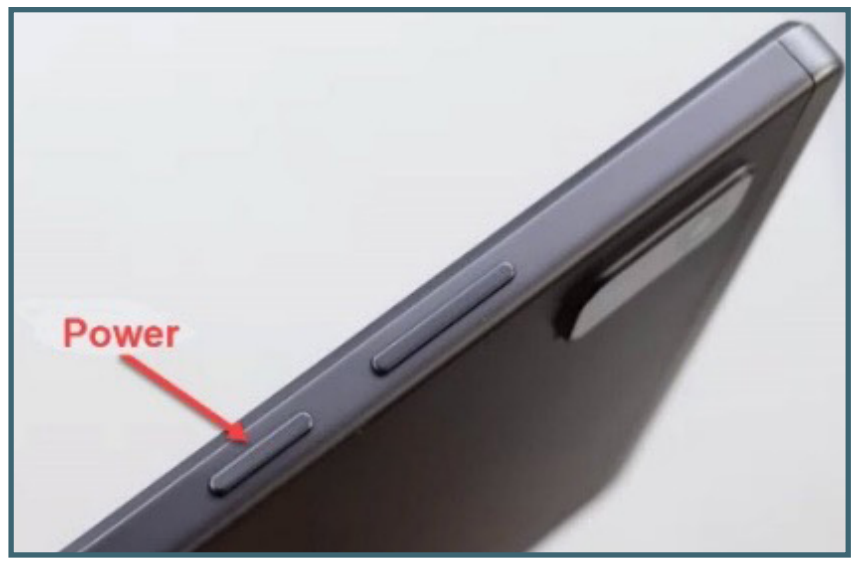

STEP 4. Launch the Fitbit app on the tablet

1. From the tablet home screen, tap the Fitbit app.
2. The Fitbit app should open to the Today screen.
3. This is the home page you will use. You do not need to navigate to any other page in the app. Please ignore the **Coach** and **You** areas of the app as they are not pertinent to the study.

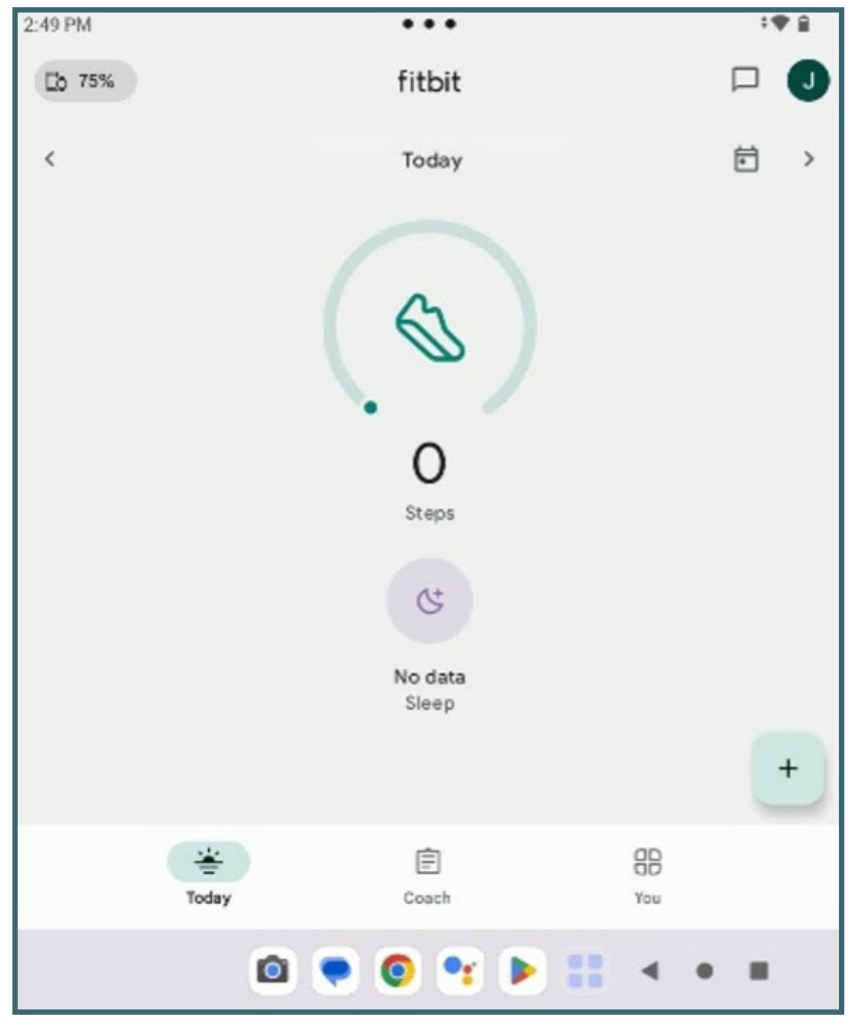

If you do happen to tap on something unintentionally, tap on the Today icon in the bottom left corner of the app to go back to the home screen.

STEP 5. Adjust your sleep schedule

The **default sleep schedule is 10:00 pm to 6:00 am.** If this **does not reflect** your sleep schedule, **update it by following these steps:**

1. Open the Fitbit app
2. Tap the Sleep icon which shows the moon and star.
3. On the sleep page, tap the three dots in the upper right corner.
4. Choose Settings
5. Go to Bedtime
6. Tap Set time
7. Enter a time.
8. Tap Set goal
9. Go to Wake up Time
10. Tap Set time
11. Enter a time.
12. Tap Set goal
13. Use the back arrow in the upper left corner to go back to the Sleep screen.
14. Use the arrow in the upper left corner to go back to the Today screen.

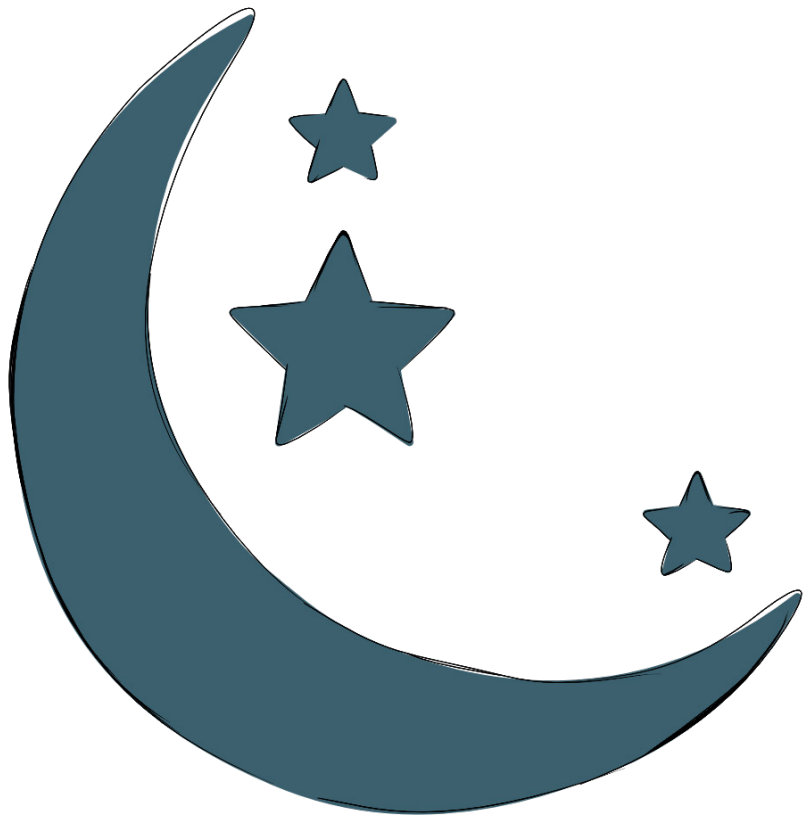

THE CLOCK SCREEN

- The home screen of the Fitbit has been configured to show a clock.
- Under the hands of the clock you will see small icons for heart rate (a heart icon), active zone minutes (three up pointing blue arrows) and steps (a shoe). On the clock screen, if you tap on the face, it will toggle through the data for heart rate, active zone minutes, and steps.
- Do not be worried if the screen appears black while you are wearing it. To preserve the battery, the device is designed to "go to sleep" throughout the day.
- Double-tap the face of the watch to "wake up" the device and see the clock again.

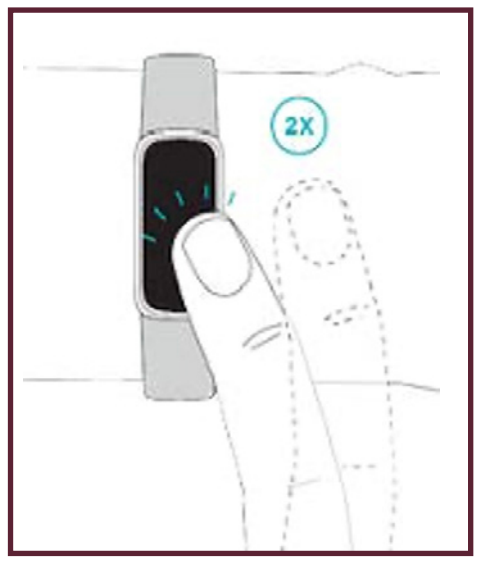

- When you wear the Fitbit inspire 3 it is easy to inadvertently swipe side-to-side or up/down on the face. To prevent any accidental changes to the settings when this happens, you can return to the clock by pressing both sides of the Fitbit at the same time.

Return to the clock screen by pressing both sides of the Fitbit simultaneously.

SYNCING THE FITBIT

- The process of "syncing" sends the data stored on the device to the Fitbit account.
- Syncing your data is an important part of this study. If you do not sync the device frequently, we may not receive all of the data the device collects.

We recommend syncing the device at least every 3 days.

The study tablet must be connected to Wi-Fi to sync and the Fitbit must be close to the tablet.

Please see each icon's purpose below :

- **View all apps** – View a list of all apps installed on the tablet
- ◀ **Go back** - Return to the previous menu or exiting an app
- ● **Home** - Return to the Home screen
- ■ **Switch apps** - View a list of open apps and switch between them or close them by clicking Clear All

CHARGING THE LENOVO TABLET

- Charge the tablet with the charger provided at least every three days. We recommend you charge the Fitbit and tablet at the same time.
- You can see the remaining battery life of the tablet in the upper right corner of the home screen.

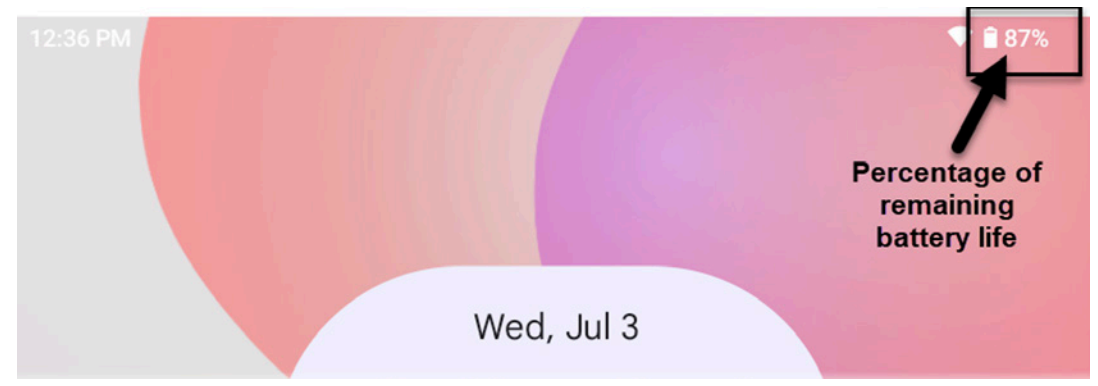

- Charge the tablet with the charger provided at least every three days. We recommend you charge the Fitbit and tablet at the same time.
- If the tablet reaches 10% battery life, a warning will appear. Please charge the tablet immediately if this occurs.

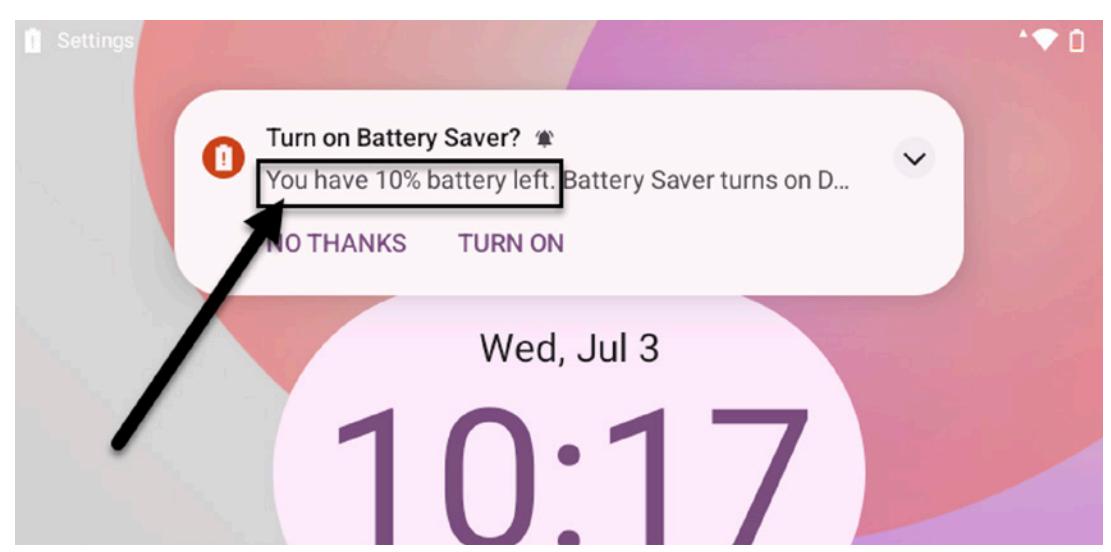

- Use only the charger included in the kit! Connect the A/C adapter to a suitable power outlet. To protect your privacy, do not connect it to a computer or laptop. The tablet may become warm when charging. Ensure that you have adequate ventilation around the charging device. If the A/C power adapter or charging cable become damaged, do not use them and contact your study coordinator for a replacement.

FITBIT INSPIRE 3

USER GUIDE

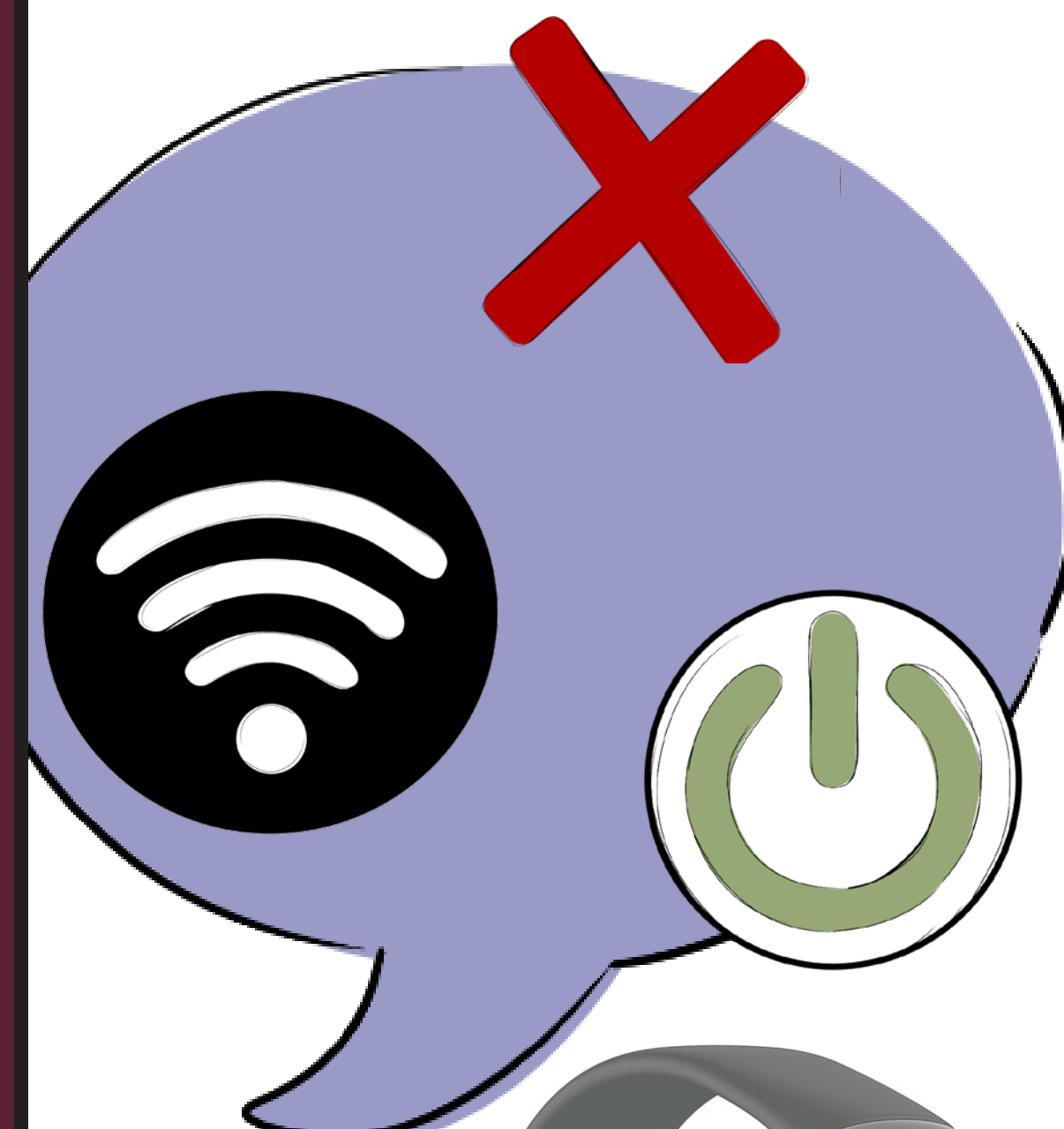

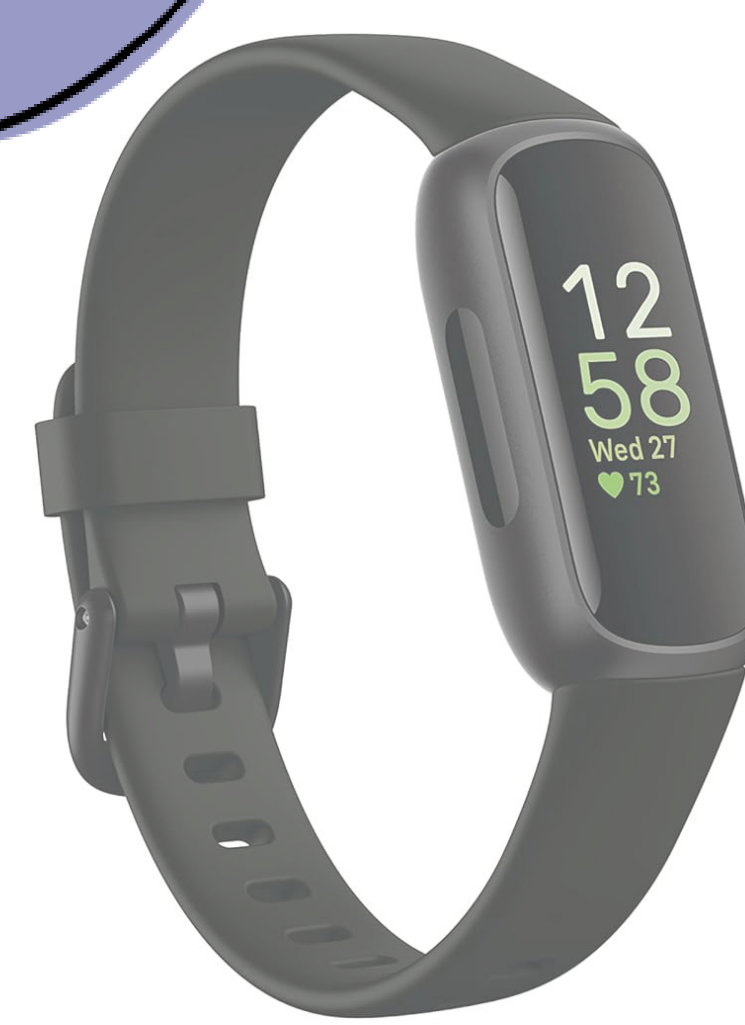

WEARABLES
PILOT STUDY

FITBIT INSPIRE 3 AND THIS STUDY

The Fitbit has been setup for you with the minimal settings that are needed for this study. To protect your privacy and to make the Fitbit as minimally invasive as possible, only those features that cannot be disabled and those that are needed for the study are enabled.

The study will collect data on your activity and your sleep so we ask that you wear the Fitbit as much as possible while you are sleeping and when you are not resting.

There is no GPS in the Fitbit. Your location is not shared.

WEARING THE FITBIT

The Fitbit is designed to be worn day and night and will collect data whenever you are wearing it. The device has been configured to disturb you as little as possible. If you meet the steps goal that has been configured, then you may feel a vibration on the wrist or see the face light up. This is normal. Other than this, you should not get vibrations from the watch.

It is recommended that you wear the band loosely enough that it can move back and forth on your wrist. During walking, try moving the band higher on your wrist (2-3 finger widths above your wrist bone) to get a better fit so it's secure, but not too tight.

Please keep the Fitbit on as much as possible.

You do not need to remove it if your hands are in water or while you shower. You may swim in this device. Dry it off to prevent discomfort or skin irritation.your wrist (2-3 finger widths above your wrist bone) to get a better fit so it's secure, but not too tight.

The Fitbit is water resistant to 50 meters for 10 minutes.

Follow these steps to sync the Fitbit at least every three days.

1. Ensure that the tablet is connected to Wi-Fi.
2. Open the Fitbit app on the tablet.
3. The Today screen will open.
4. With your index finger, swipe down on the center of this screen.
5. A banner will appear at the top to indicate that the device is syncing.

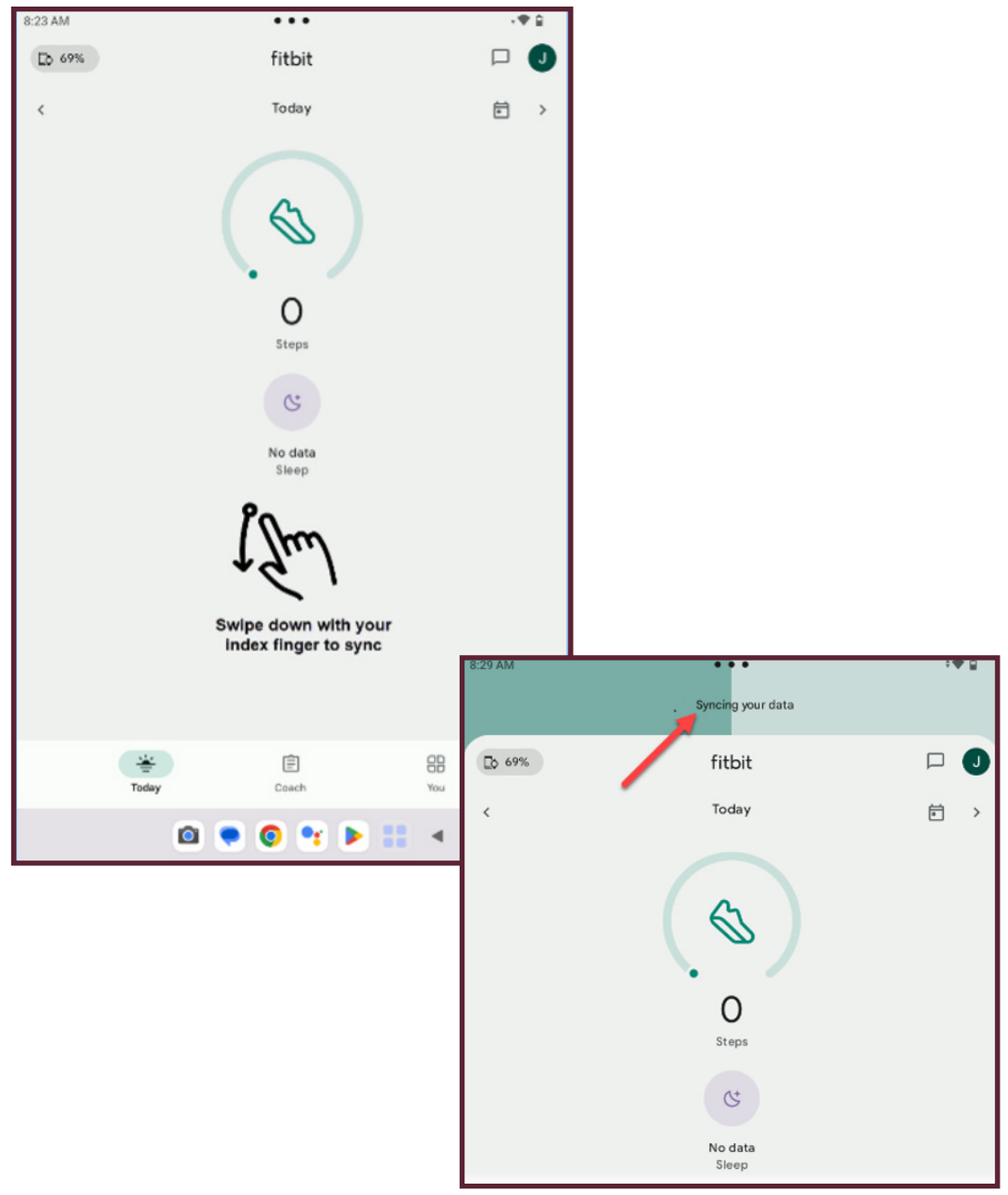

CHARGING THE FITBIT

- To check the battery life on the Fitbit while you are wearing it, if the watch face is black, double tap it to turn it on. Then swipe up once you should then see the battery icon with the percentage level. Press the sides of the device at the same time to bring the clock screen back.
- The Fitbit must be charged throughout the duration of the study. It takes about 1 to 2 hours to fully charge the device from a completely drained battery. Do not wait until the battery is drained before you charge.
- Because the study is looking at your activity and sleep, you should not charge the device while you are sleeping. The best time to charge the battery is at a time when you are resting – when you are reading, watching television, doing computer work, etc.

Try not to let your device get below 50%

Charge at least every three days

Do not charge the Fitbit

- When the Fitbit is plugged in and charging, the screen will show the battery icon. A fully charged watch shows a solid battery icon and indicates 100%.
- Please use the A/C power adapter provided to charge the Fitbit. To protect your privacy, do not connect it to a computer or laptop.

ABOUT THE LENOVO TABLET

- The tablet in the kit has been setup for the study and will be returned to the clinic. To help protect your privacy and to prevent any unintended issues, please do not use the tablet for anything other than the study.
- The tablet's home screen has a shortcut to the Fibit app and to the surveys you will complete. At the bottom of the home screen you will see a set of navigation icons. You may occasionally need to use these icons to navigate the tablet.

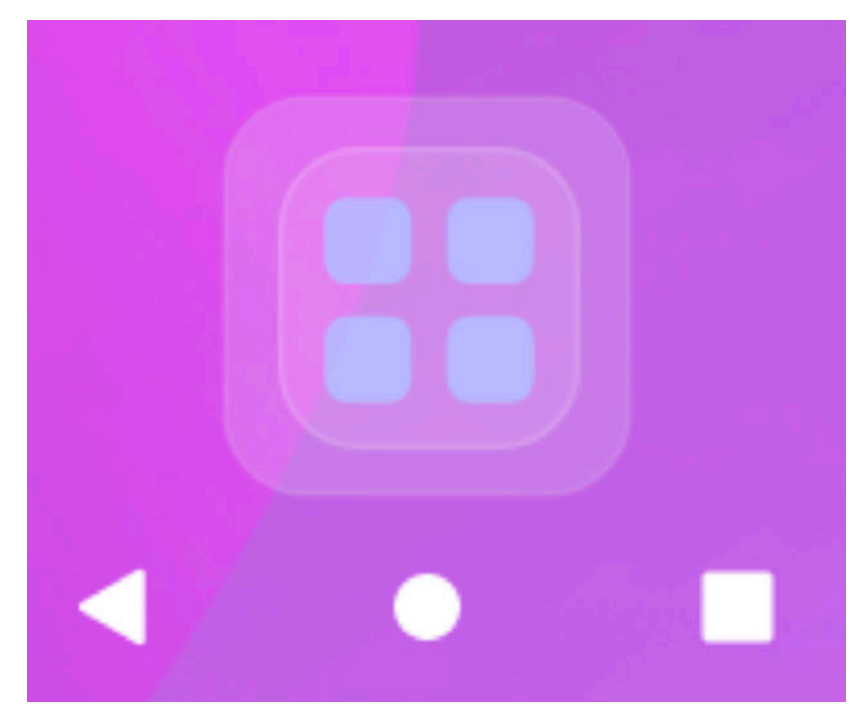

Supplement 3: Example of Frequently Asks Questions Document for One of the Three Fitness Watches Used in the Pilot

THE TABLET SEEMS UNREPONSIVE

1. Ensure that the tablet is charged.
2. Hold down the Volume down button AND the power button for 8 seconds.
3. The tablet screen may display "preparing to force shut down" before going black.
4. Wait a minute and then press the Power but-ton until you hear a vibration sound the Lenovo logo appears.
5. Allow the tablet to restart
6. When you see Unlock for all features and data displayed at the top, swipe up to see the PIN pad
7. Enter the tablet pin and tap the right pointing arrow.
8. Tap the Fitbit app on the tablet home screen to open it.

I SIGNED OUT OF THE FITBIT APP; HOW DO I SIGN BACK IN?

1. Tap the Fitbit app on the tablet home screen
2. Tap Sign in with Google.
3. Tap Continue as [name].
4. Google will log you in and the permission screen will open.
5. Use the X in the upper left corner to close out of the Permission Request screen. Do not tap SET PERMISSIONS!
6. If a screen with the heading, Fitbit needs location for some features is displayed,tap CONTINUE WITHOUT LOCATION.
7. When Are you sure? is displayed, select ALLOW LATER.
8. If any tiles for Health Connect appear, close them out with the X.
9. Sync your device. (see the Fitbit Inspire 3 usage guide in this kit)

SYNCING NOT WORKING

If your device doesn't sync, there are **three sets of steps to** perform before attempting to sync again.

Verify there is a Wi-Fi connection

1. In the status bar on the home screen, tap the icon that looks like a square with four squares.
2. Tap Settings

WHEN I WEAR THE FITBIT, DOES IT KNOW MY LOCATION?

No, it does not.

Fitbit inspire 3 does not have built in GPS tracking. Furthermore, the Fitbit is being linked to a Lenovo tablet which does not have cell service and is configured to have locations services turned off. Even if you take the Lenovo tablet out of your house to access Wi-Fi, **the settings on the tablet will prevent location information** from being acquired by the Fitbit app.

If you have any additional questions or issues, please contact the study coordinator at your site for help.

FITBIT INSPIRE 3

FAQs

& TROUBLESHOOTING

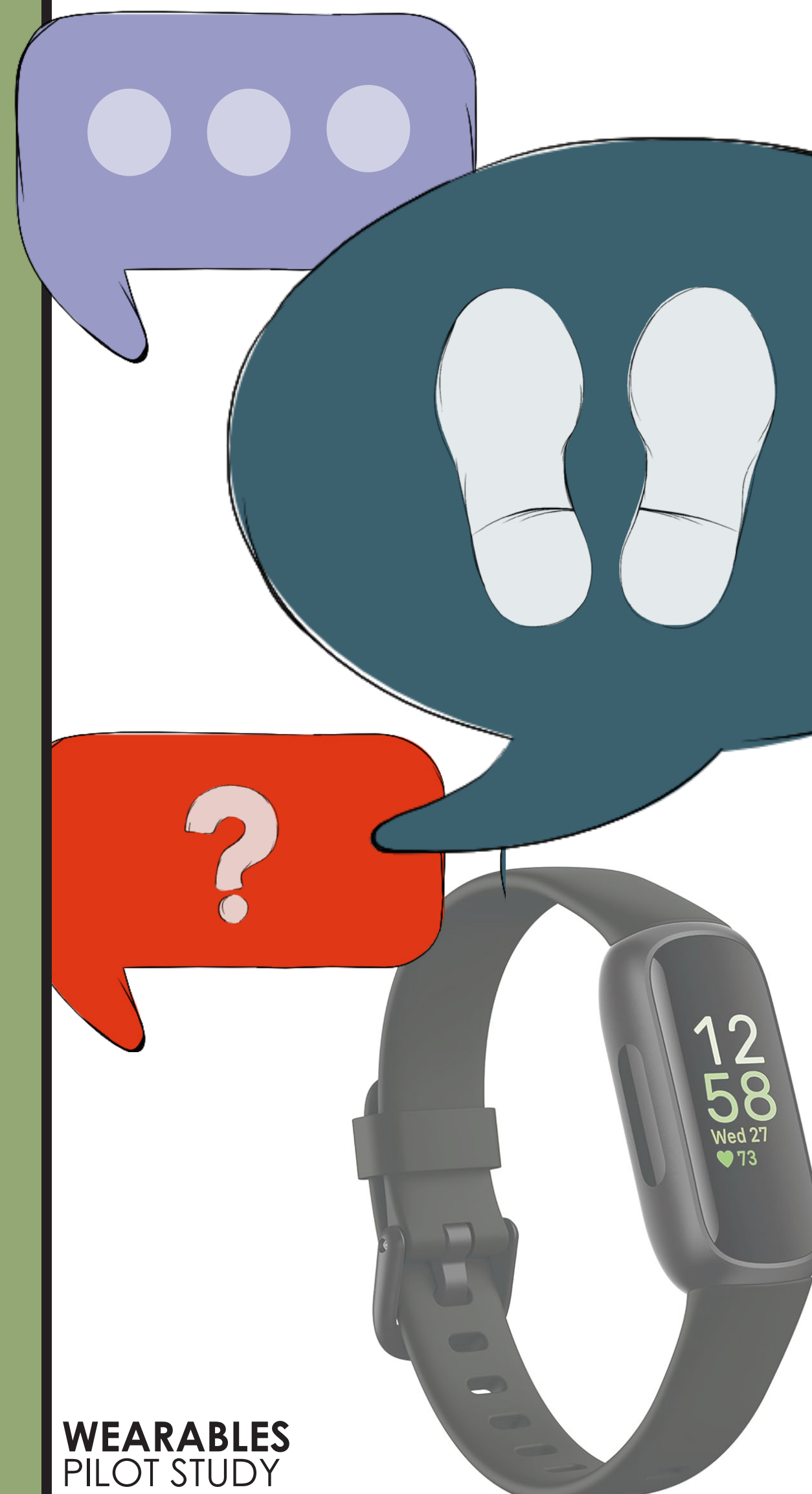

WEARABLES
PILOT STUDY

HOW DO I TURN ON THE TABLET?

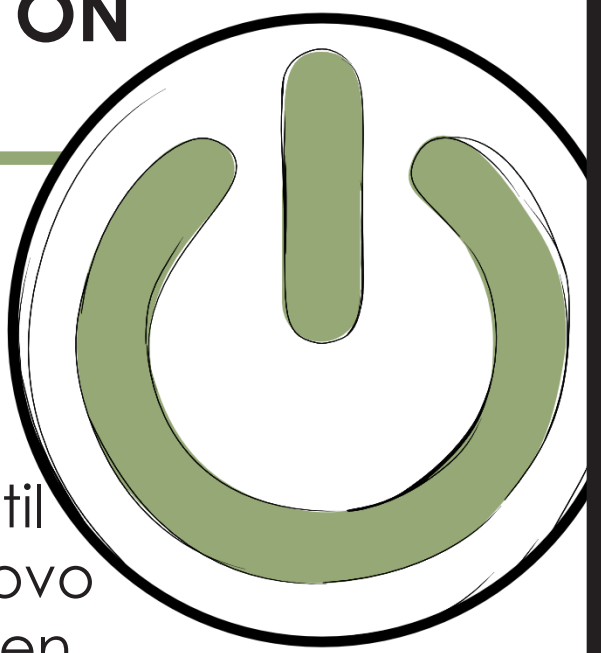

- Press and hold the **Power** button for approximately 3 -5 seconds or until you see the Lenovo logo on the screen.

HOW DO I OPEN THE FITBIT APP?

- If you close the **Fitbit** app and need to reopen it, the link is on the home screen of the tablet.
- Tap the Fitbit icon. The app should open to the Fitbit **Today** screen.

THE FITBIT APP SEEMS UNRESPONSIVE?

If you tap the buttons on the app and **nothing happens, you will need to restart the app:**

1. Tap on the square icon at the bottom of the tablet home screen.
2. Tap Clear all to close any open apps.
3. Restart the Fitbit app by tapping the Fitbit icon on the tablet home screen.

If **problems continue** to exist **follow these instructionsto** force shut down the app:

1. Press the circle icon at the bottom of the table screen to go back to the tablet home screen.
2. Long tap on the Fitbit icon. (Long tap mean you leave your finger on the app icon until you see a menu come up.)
3. Tap the information icon which is an 'i' in a circle.
4. Tap FORCE STOP.
5. Tap OK to the force stop confirmation message.
6. Tap Open to restart the Fitbit app.

3. Make sure the name of the Wi-Fi network appears under the Network & internet heading
4. If it does not appear Tap Network & internet and reconnect to Wi-Fi using the instructions in [Getting Started
5. Go back to the Fitbit app on the tablet and try to sync again.

Verify Bluetooth is turned on

1. In the status bar on the home screen, tap the icon that looks like four squares.
2. Tap Settings
3. Make sure On is listed under the Bluetooth heading.
4. If it displays Off, switch the Bluetooth back on.
5. Go back to the Fitbit app on the tablet and try to sync again.

Restart the Inspire 3 watch

1. Tap twice on the clock face to turn on the display
2. Swipe down once and then scroll slowly until it says SETTINGS.
3. Tap SETTINGS.
4. Swipe up slowly until it says Restart Device
5. Tap Restart Device
6. An information message will be displayed. Read this and scroll down until you see the bright blue button labeled RESTART
7. Tap RESTART.
8. The watch will go black for a moment and then the Fitbit logo will appear. It should take

Go back to the Fitbit app on the tablet and try to sync again.

HOW CAN I SEE MY DATA?

- Your data is viewable on the Fitbit app.
- Use the forward and back arrows on the Today screen to view past and current day's data. The calendar icon brings you back to the Today screen.
- Tap the steps icon to see a breakdown of the number of steps recorded by hour.
- Tap the sleep icon to see sleep duration and sleep stages that were recorded.

HOW CAN I HIDE MY SLEEP OR STEPS?

1. Open the Fitbit app.
2. Make sure it says Today at the top of the screen. If it does not, use the arrows to go to today or tap the calendar icon.
3. Tap the pencil icon in the upper right corner of the screen.
4. Tap the pencil in the Custom preset tile.
5. Tap on Metric #2.
6. Tap Off.
7. Tap Save.
8. Tap on Metric #1.
9. Select the metric under the Activity heading you would prefer to see. You must show at

HOW CAN I ADJUST THE WATCH BRIGHTNESS?

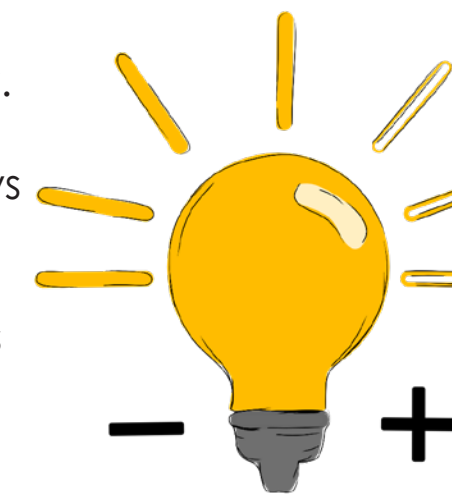

1. Tap twice on the clock face to turn on the display.
2. Swipe down once and then scroll slowly until it says SETTINGS with a gear icon.
3. Tap SETTINGS.
4. Swipe up slowly until it says DISPLAY SETTINGS.
5. Tap DISPLAY SETTINGS
6. Tap BRIGHTNESS
7. The options are Normal, Dim and Max
8. Tap until the desired setting is displayed, then press the two buttons on the side simultaneously to go back to the clock face.

The brightness should be changed to what you selected.

HOW CAN I INCREASE/ DECREASE THE STEPS GOAL?

1. Open the Fitbit app on the tablet
2. In the upper right corner, tap on the initial that appears in the dark green circle
3. Tap on Fitbit Settings
4. Under Preferences, tap on Activity
5. Under Daily Activity, tap on Steps
6. Use the +/- buttons to increase or decrease the steps goal
7. Tap Set goal
8. Use the back arrow in the upper left corner of the app to go back to the Today screen

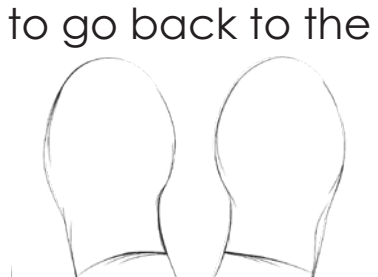

Supplement: Supplementary file 1 [file Supplementaryfile1.pdf]
