# Supplementary material for: Acceptability and feasibility of fitness watches to monitor sleep and physical activity among women living with HIV: broadening the approach to optimizing cardiovascular health
Source: Front Digit Health. 2026 Sep 3;8:1881771. doi: 10.3389/fdgth.2026.1881771 (PMC13582592; doi:10.3389/fdgth.2026.1881771)
Supplement: Supplementary file 2 [file Table1.docx]

**Supplemental Table 1**: Summary of Self-Reported Data and Fitness Watch Data on Physical Activity Measures

|  | | Assigned Fitness Watch | | |  |
| --- | --- | --- | --- | --- | --- |
| Physical Activity Characteristics | | **Inspire 3**  **(N=10)** | **Vivofit 4**  **(N=10)** | **Vivosmart 5**  **(N=10)** | **Total**  **(N=30)** |
| **From Self-Report:** | |  |  |  |  |
|  | Number of the 10 days with at least 1 hour of moderate activity | 5.0 (3.6) | 3.6 (4.1) | 5.7 (3.6) | 4.8 (3.8) |
|  | Number of the 10 days of at least 30 minutes of vigorous activity | 3.2 (3.1) | 2.0 (3.0) | 4.1 (4.2) | 3.1 (3.5) |
|  | Number of naps taken over the 10 days | 1.4 (2.6) | 5.2 (5.8) | 1.9 (2.1) | 2.8 (4.1) |
|  |  |  |  |  |  |
| **From Fitness Watch:** | |  |  |  |  |
|  | Average minutes total activity | 292.8 (115.9) | 60.8 (28.1) | 171.4 (58.0) | 175.1 (123.1) |
|  | Average resting heart rate^1^ | 66.4 (15.8) | NA | 66.0 (7.6) | 66.2 (12.3) |
|  | Average daily steps | 9,019 (4,113) | 5,777 (2,896) | 7,477 (1,717) | 7,422 (3,295) |
|  | Average daily distance (miles) | 3.6 (1.7) | 2.3 (1.2) | 3.1 (0.7) | 3.0 (1.3) |

^1^Heart rate not measured on Vivofit 4 device
